# Year of Expanding into Circulating Biomarkers

**DOI:** 10.5772/60126

**Published:** 2015-01-29

**Authors:** Shidong Jia, Winston Patrick Kuo

**Affiliations:** 1 Genentech Inc., South San Francisco, USA; 2 IES Diagnostics, Inc., Cambridge, USA

**Keywords:** Extracellular vesicles, exosomes, microvesicles, circulating tumour cells, cell-free circulating DNA, editorial

## Abstract

This editorial article summarizes the achievements and current challenges for the *Journal of Circulating Biomarkers* (JCB) regarding a more strategic approach to branding and attracting a high quality variety of articles. More emphasis is placed on fostering engagement with academic and industry sources operating at the cutting-edge of translational technologies applied to the field of circulating biomarkers (interface between extracellular vesicles including exosomes and microvesicles, circulating tumour cells, cell-free circulating DNA and circulating protein markers) and with those in the investment arena seeking and providing private funding for this area of research.

We are proud to announce that during the past year we have experienced several ongoing developments, one of which has been the expansion of our editorial board with key opinion leaders in the circulating biomarker field including Yvon E. Cayre (Pierre and Marie Curie University, France), Alain Charest (Tufts University School of Medicine, USA), Yong Chen (Ecole Normale Superieure, France), Daniel Haber (Harvard Medical School, USA), Pavel Laktionov (Russian Academy of Sciences, Russian Federation) and David T. Wong (University of California Los Angeles, USA), all of whom contribute as part of our associate editor's team. Additionally, Aled Clayton (Cardiff University, United Kingdom), Aleksandra Fučić (Institute for Medical Research and Occupational Health, Croatia), Stefan Holdenrieder (University Hospital Bonn, Germany), Hongjun Kang (Chinese PLA General Hospital, China), Ming-Lin Liu (University of Pennsylvania, USA), Jan Lotvall (Göteborgs Universitet, Sweden), Jie Ma (Fudan University, China), Pierre-Yves Mantel (Harvard School of Public Health, USA), Dario Marchetti (Baylor College of Medicine, USA), P. Shannon Pendergras (Ymir Genomics, USA), Eva Rohde (Paracelsus Medical University, Austria), An Song (Genentech Inc., USA), Matthew J. Wood (University of Oxford, United Kingdom), Bruce R. Zetter (Harvard Medical School, USA) and Hanyu Zhu (Chinese PLA General Hospital, China) serve as members of our editorial board.

In 2014, we published high quality papers covering research topics in the area of cell-cell communication via exosomal heat-shock proteins, the function of erythrocyte-derived extracellular vesicles in malaria as it pertains to understanding their role in immune regulation and cell-cell communication, the development of stem cell-derived exosomes as a cell-free regenerative medicine, as well as the detection of human c-*Myc* and *EGFR* amplifications in circulating extracellular vesicles in mouse tumour models. We also published a paper on circulating free DNA and the influence thereof following lung parenchyma surgical manipulation.

In addition, we are pleased to have established a partnership/collaboration with the BioPharma Research Council (http://www.biopharmaresearchcouncil.org/), spearheaded by its Executive Director, Joanne Gere. The BioPharma Research Council has been developing a vibrant community for scientists as they navigate today's complex science environments. Our unique resources – including close relationships with leading companies and suppliers – and agile approaches have established recurrent opportunities for breakthrough discussion and debate throughout the drug discovery process and delivery pipeline. This partnership/collaboration resulted in the establishment of a short course titled, “Extracellular Vesicles: The Transition from Tissue to Liquid Biopsies”, which was conducted as a four-session webinar series in October and November 2014. The goal of the course was to provide, at an introductory level, an exchange between researchers from within academia and industry pertaining to the characterization of the applications of EVs in clinical and translational research, eventually to be implemented within clinical practice. We were able to utilize our editorial team to present topics that included: “Overview of Extracellular Vesicles” by Jan Lötvall, MD, PhD; “Clinical Diagnostic Applications of Extracellular Vesicles” by Johan Skog, PhD; “Commercialization Aspects of Extracellular Vesicles” by Alexander “Sasha” Vlassov, PhD; “Extracellular Vesicles: Therapeutic Hurdles” by Eva Rohde, MD; “Funding Opportunities for Extracellular Vesicle Research” by Angel Ayuso Sacido, PhD. A meeting dispatch was published online and is available on the JCB website (http://www.intechopen.com/journals/journal-of-circulating-biomarkers/short-course-in-extracellular-vesicles-ndash-the-transition-from-tissue-to-liquid-biopsies). From this experience, we learned that coordinating the preparation process of such webinars required approximately six months. We plan to continue several webinar series on circulating tumour cells and cell-free circulating DNA later this year.

Finally, we launched a special issue titled, “The Biology and the Clinical Utility of Circulating Tumor Cells”, with a focus on detecting the latest findings in CTC research and clinical value. More details are located in the news section of the *Journal of Circulating Biomarkers* (http://www.intechopen.com/journals/journal-of-circulating-biomarkers).

As we continue to strive to publish the best scientific research in all fields related to circulating biomarkers, the editorial team aims to focus on additional topics such as cell free DNA and disruptive technologies, among others.

JCB accepts original and review articles, as well as editorials, perspectives, short research reports, protocols and methods, notes to the editor, letters to the editor and meeting dispatch reports. We would also like to mention that InTech Publishing have decided that JCB will not apply any article processing charges for authors whose research papers have been accepted for publication in Volume 4/2015 of the journal. All manuscripts are peer-reviewed for scientific quality and the review process is conducted entirely online. The editorial manager facilitates the manuscript processing time, thereby reducing costs and providing a better experience to both our authors and reviewers. We have received numerous positive comments about this evolving electronic system and are indebted to InTech for managing this programme for us. For more information, please review our new manuscript submission system at http://www.editorialmanager.com/exo/default.asp. In addition, we are expediting the acceptance to publishing time for our authors and will request informal opinions from our editorial board for borderline cases.

JCB will continue to partner with scientific societies and academic and industry leaders in order to advance the field and to allow free access to knowledge in the area of circulating biomarkers.

We gratefully acknowledge the editorial team and publishers for making the continued success of the journal possible.

